# "A mathematical theory of evolution": phylogenetic models dating back 100 years

**DOI:** 10.1098/rstb.2023.0297

**Published:** 2025-02-20

**Authors:** Noah A. Rosenberg, Tanja Stadler, Mike Steel

**Affiliations:** ^1^ Department of Biology, Stanford University, Stanford, CA, USA; ^2^ Department of Biosystems Science and Engineering, ETH Zürich, Basel, Switzerland; ^3^ SIB Swiss Institute of Bioinformatics, Lausanne, Switzerland; ^4^ Biomathematics Research Centre, University of Canterbury, Christchurch, New Zealand

**Keywords:** mathematical models, phylogenetics, stochastic processes

## 1. Introduction

Charles Darwin’s 1859 *Origin of Species* [1] famously contained only a single figure: a schematic depiction of a phylogenetic tree. In several pages of accompanying text that amounted to an extended caption for his tree figure, Darwin explained how the tree could both represent the descent of biological lineages and provide a scheme for taxonomic grouping:

*The limbs divided into great branches, and these into lesser and lesser branches, were themselves once, when the tree was small, budding twigs; and this connexion of the former and present buds by ramifying branches may well represent the classification of all extinct and living species in groups subordinate to groups.* [1, p. 129]

Darwin’s description of his tree of life appeared almost simultaneously with another event that would later become a milestone in phylogenetics: Arthur Cayley’s 1857 publication of 'On the theory of the analytical forms called trees'*,* perhaps the first effort to define trees as mathematical objects in graph theory [2]. In seeking to describe the sequences in which strings of mathematical operators can be applied, Cayley made a connection between tree structures and strings of symbols. A version of this idea persists in phylogenetics as the basis for the Newick notation to represent evolutionary relationships.

The temporal proximity of Darwin’s tree figure and Cayley’s mathematical description of trees is a tantalizing historical juxtaposition. Cayley’s article was not far from Darwin’s orbit; the paper that immediately followed it in the pages of *The London, Edinburgh, and Dublin Philosophical Magazine and Journal of Science* was a letter from ‘the Rev. Prof. Sedgwick, M.A., F.R.S. &c’—Darwin’s geology teacher and correspondent, Adam Sedgwick [3]. Yet no major link between evolutionary trees and mathematical trees would be made for many decades. Darwin himself was not a mathematical researcher, having written in his autobiography:

*I attempted mathematics, and even went during the summer of 1828 with a private tutor (a very dull man) to Barmouth, but I got on very slowly. The work was repugnant to me … and in after years I have deeply regretted that I did not proceed far enough at least to understand something of the great leading principles of mathematics* … [4, p. 58]

The interpretation of the evolutionary descent of species as a mathematical tree-like process would emerge only from the publication in 1925 by George Udny Yule of a much-celebrated article [5], *A mathematical theory of evolution, based on the conclusions of Dr J. C. Willis, F. R. S*. Yule, who lived from 1871 to 1951, was an intellectually broad scholar known for valuing his freedom to work across many areas [6]—a ‘loafer of the world’ in Yule’s own phrase [7]. Trained first in engineering and experimental physics, he became a statistician just as the field of statistics was emerging, and he is remembered as one of its pioneers [8,9]. Yule is noted as an important contributor to the beginnings of quantitative social science; he had an interest in economic and population statistics and was early to the project of formulating and testing social science hypotheses statistically [10]. He worked on a number of biological science applications, including an early 1902 contribution to the effort to reconcile Mendel’s newly rediscovered laws of particulate inheritance with the often-continuous nature of phenotypic variation [9,11].

A friendship with the botanist John Christopher Willis [7, p. 8] led to the seminal 1925 article. As described by Pennell and MacPherson in this themed collection [12], Willis proposed a hypothesis that linked the age and area of species. Recognizing the statistical nature of Willis’s hypothesis, Yule formulated a mathematical model of the sequential bifurcation of biological lineages, seeking to understand its predictions about the relationship between the size of a genus and its age. We now regard the tree-like descent model implied by the Yule process as a pioneering model of mathematical phylogenetics—but among Yule’s contributions to contingency tables, correlation, regression, time series analysis, statistics for epidemiology, literary attribution and population science, and a widely used early statistics textbook, it occupies a small place in his overall oeuvre [7]. Although the Yule process soon went on to have a life of its own in probability and stochastic processes [13], in line with Yule’s ‘loafer of the world’ approach of pursuing statistical problems in many applied areas, he did not develop a programme of further evolutionary research building on the article; its monumental effort of new mathematics, detailed tedious calculation and dozens of figures and tables would stand on its own in Yule’s work.

Curiously, as Lambert comments in his article in the themed collection [14], although the connection of what we now know as Yule’s birth process to tree-like branching is second nature to a modern reader, Yule did not view his model of the bifurcating descent of species and genera in terms of evolutionary trees, instead focusing on its use for counting species within genera and relating the size of a taxonomic group to its age. It would be several more decades before the study of trees evolving in time would merge with mathematical models of trees in the 1960s and 1970s—in multiple contexts, including palaeobiology, phylogenetic inference, population genetics and urn models in probability—solidifying in the 1990s as a mature field, with Yule’s 1925 paper [5] recognized as a prescient founding document that anticipated many challenges that persist into the modern area of mathematical phylogenetics [12,14].

A modern reader will find much to enjoy in Yule’s paper. To a modeller, the paper resonates in its perspective that the mathematically simplest choice is often suitable in the absence of empirical support for alternatives; in its use of a time scale natural for the model rather than a scale of years, a now familiar idea in molecular evolution and coalescent theory; and in its deliberate approach to communicating results to less mathematically oriented readers ('I will now endeavour to summarize the conclusions reached in general terms which I hope may be comprehensible to the non-mathematical biologist' [5, p. 25]). Yule approaches this last task by opening the paper with several pages of analysis of the biological significance of the findings, only afterwards introducing the mathematics that undergirds the claims. Juxtaposed with its modernity, the paper has delightful archaisms, such as the idea that a mutation does not simply occur but is ‘thrown’ from an ancestral group to a descendant. One also encounters reminders of just how much was unknown in 1925—for example, the timing of geologic time periods, needed by Yule for his numerical estimates [5, p. 76].

This themed collection—on the occasion of the 100th anniversary of Yule’s often unusually modern article, largely lost to evolutionary biology for decades—explores the science of mathematical phylogenetic modelling: modelling that focuses on the stochastic divergence processes of biological lineages and on recovering features of those processes from the lineages that they produce. Three articles in the collection focus on the historical significance and legacy of Yule’s paper. The rest of the collection is divided into sections covering (i) mathematical developments on phylogenetic models, (ii) statistical methods based on the models, and (iii) applications to diverse areas of biology, from macroevolution to epidemics and immunology.

## 2. The papers in the themed collection

### (a) Background and legacy of Yule’s paper

The collection begins with a close reading of Yule’s paper by Pennell & MacPherson [12] in relation to macroevolution, the topic that it originally sought to address. They describe the debates about speciation that were taking place when J. C. Willis proposed his hypothesis connecting a broader geographic range to older species age. They cover Yule’s recognition that Willis’s hypothesis could be approached by a probabilistic model, recapitulating his original derivation of the distribution of the number of species in a clade as a function of the birth rate and the length of time since the first bifurcation—before contrasting it with more modern derivations. Pennell & MacPherson note that Yule’s rejection of Willis’s hypothesis can be viewed as a finding that stochasticity alone can explain the pattern of clade sizes, previewing the important role that later researchers would find for chance in macroevolution.

A long section of the commentary by Pennell & MacPherson describes the excitement in the field of palaeobiology upon the rediscovery of macroevolutionary models in the 1970s and the emerging acknowledgement of Yule’s early contribution. The paper concludes with remarks on three topics that Yule’s paper anticipated: (i) the potential for models to consider diversification at different hierarchical levels, (ii) the challenge of reconciling mathematical models with variable practices concerning the taxonomic level to which groups of organisms are assigned, and (iii) the modelling of the within-species population processes that underlie decisions about speciation parameters. Commenting on the prescience of the paper, Pennell & MacPherson write:

*Reading Yule’s paper today is a jarring experience. While the presentation of the work is certainly consistent with that of his time, the style of analysis and broader way in which he thinks of the problem seems right at home in the macroevolutionary literature of the late twentieth century.*

Lambert [14] provides further historical insight into the central results in Yule’s paper, highlighting some lesser-known aspects, and detailing the various ways in which Yule’s results have been overlooked, rediscovered and sometimes misinterpreted in the following decades. Lambert provides a precise description (in modern language) of Yule’s frequency distribution for the sizes of genera, then extends these results in new directions. A highlight is a precise stochastic analysis of the distribution of the triples of coalescent times, ages and sizes of genera in the main theorem of the paper (Theorem 3.2), using coalescent point process theory. Two further propositions describe conditions under which long-tail distributions (of the type Yule identified) might be expected under two time-homogeneous stochastic settings (linear birth–death process with constant rates, and a pure-birth process with singleton jumps). Viewing Yule’s model as an urn scheme, Lambert compares and contrasts its predictions with those of two other urn schemes: the Hoppe and Simon urns. By extending the Yule urn model in two ways (tied to the settings of the two previous propositions), the paper presents conditions under which a long tail arises in the frequency distribution of urn sizes.

The review by Tavaré [13] begins with a concise summary of the stochastic properties of linear birth–death processes, and the trees they generate (noting the distinction between the *complete* tree and the *reconstructed* tree). The author then focuses on the impact of immigration processes in this setting, an extension that dates back to Kendall in the 1940s. This leads to the celebrated Ewens Sampling Formula for the counts of the number of families of given sizes, conditional on the total population size at a given time. A second application—to an ecological process considered by R. A. Fisher—is then presented through a more recent lens. In the final part of his paper, Tavaré shows how approximate Bayesian computation can be applied to birth–death processes that model cell populations, with the aim of deriving posterior distribution estimates of mutation and split rate parameters.

### (b) Mathematical phylogenetic modelling

The next group of papers delves into combinatorial aspects of trees. Probabilistic tree models are associated with spaces of possible trees, often involving discrete sets of trees along with associated sets of continuous branch lengths. Analyses that consider stochastic aspects of the discrete structure are important for understanding tree features of biological interest.

The review by Steel [15] examines the impact of different stochastic models on various aspects of tree shape. Explaining tree balance is topical since it is well known that most birth–death models generate phylogenies that are typically more balanced than the ones biologists typically reconstruct from data (at the other extreme, the uniform distribution of phylogenetic trees is overly imbalanced). The paper then focuses on mathematical approaches to a question in biodiversity conservation: can we predict biodiversity loss due to rapid extinction at the present? Various measures of biodiversity are possible, and a simple but widely used one is Dan Faith’s ‘phylogenetic diversity’ (PD) measure. The loss of PD on birth–death trees under extinction at the present is reviewed, and then compared with a more recent approach that considers the loss of underlying features associated with the loss of PD, where a model of discrete feature gain and loss is superimposed on the branches of the tree. The two models lead to similar (but not identical) predictions, which can be described by explicit formulae.

Considering only the discrete structure of trees, a review by Fuchs [16] explains an equivalence between the probability model induced on discrete tree structures by the Yule birth process—often termed the Yule, Yule–Harding or Yule–Harding–Kingman model for tree shape—and the *random binary search tree* model in computer science. In particular, the binary search trees constructed from a uniform distribution of permutations of {1,2,…,n−1} can be placed in correspondence with the uniform distribution of *labelled histories*, the possible sequences of branching events that give rise to n labelled leaves. As random binary search trees have been studied in detail in investigations of the running time of algorithms, methods from theoretical computer science can be used to derive corresponding results in phylogenetics. A useful concept is that of the *additive shape parameter*, in which a function of a tree is obtained as a sum of three quantities: the function computed on its left subtree, the function computed on its right subtree and a quantity computed at its root. Fuchs provides examples of mathematical phylogenetics results that can be derived using this concept, including properties of several indices associated with tree balance—the Sackin index, the cherry index and the cophenetic index.

Dickey & Rosenberg [17] perform a combinatorial study of labelled histories, expanding beyond bifurcating trees, as in the Yule birth process, to multifurcating trees. Supposing that each internal node of a tree possesses exactly r child nodes, Dickey and Rosenberg conduct a variety of enumerative studies of labelled histories for multifurcating trees. In particular, they count the r -furcating labelled histories across all labelled tree topologies with a specified number of leaves and the r -furcating labelled histories that can be associated with a specific labelled tree topology. They also count r -furcating labelled histories in settings that allow branching events to occur at the same time—providing a recursion for enumerating labelled histories in settings that extend the classic scenario with *two* generalizations: multifurcation and simultaneity. The new directions launched by this study suggest a number of open problems.

The paper by Chauve *et al.* [18] continues the combinatorial investigation of trees (as well as a class of phylogenetic networks). The authors use a novel way of encoding rooted phylogenetic trees to describe a tree rearrangement operation (the ‘HOP’ operator), which in turn provides a new way to measure distances between phylogenetic trees. Unlike most existing tree metrics based on rearrangement operations (e.g. nearest-neighbour interchange, subtree-prune-and-regraft, tree-bisect-and-reconnect), which are typically NP-hard to compute, the new metric has the remarkable property of being computable in near-linear time and so is applicable to large datasets. The authors compare their metric with other existing ones, and show how it can be extended beyond trees to the class of tree–child networks.

Moving beyond phylogenetic trees, a review by Bienvenu [19] provides an overview of various ways that probabilistic techniques can be applied to study random phylogenetic networks. By focusing on random network models that are mathematically tractable, the paper describes how stochastic ideas can be used to enumerate and sample the networks, complementing more traditional combinatorial approaches such as asymptotic enumeration. Properties of large trees and networks can also be derived using probabilistic approaches, such as the limiting distribution of the Sackin and $B_{2}$ balance statistics. In addition to standard probabilistic techniques (method of moments and the Stein–Chen method), the paper points to the potential in more recent approaches, such as the notion of viewing certain classes of random phylogenetic networks as ‘blowups’ of Galton–Watson trees. Bienvenu describes two promising approaches for investigating the ‘geometry’ of large phylogenetic networks.

### (c) Statistics and inference methods under phylogenetic models

Four papers consider statistical analyses under birth–death models. A review by Rannala & Yang [20] studies the inference of the speciation and extinction rates of a birth–death process itself. One generalization of a birth–death process, the ‘generalized birth–death model,’ allows the speciation and extinction rates to vary over time, maintaining the assumption that the rates are shared by all extant lineages. Rannala & Yang consider the question of identifiability, whether it is possible in principle to infer the speciation and extinction rates over time from the count of the number of lineages present in the tree measured over time. Reviewing recent results, they compute probabilities of various outcomes under the generalized birth–death model, showing that the model is not identifiable, in that multiple values of the parameter vector produce the same probability of the lineage-through-time data. Rannala & Yang also discuss modified versions of the model to allow piecewise constant rather than arbitrarily varying rates. In this relaxed version of the model, the parameters are more constrained, and identifiability is achieved.

Focusing on discrete tree shapes, Kersting *et al.* [21] study the properties of tree balance statistics under various probabilistic models of tree shape. Each statistic can be calculated from the unlabelled shape of a tree; treating the Yule model as the null model for tree shape, using simulations under an alternative model, they calculate the power of each of the statistics to reject the null. The study tabulates many statistics and alternative models, performing its analysis in diverse conditions. Kersting *et al.* [21] find that while trends are sometimes observable, no single statistic consistently has higher power across the array of settings that they consider. The paper suggests that numerous tree balance statistics will remain relevant in empirical problems, offering software to facilitate their continued use.

The Yule model for tree shape emerges in the classic Kingman coalescent in population genetics. The paper by Zhang & Palacios [22] explores extensions of the Kingman coalescent to the Λ -coalescent, which allows for multiple mergers of lineages (rather than just pairwise mergers). By focusing on the Λ -coalescent model with an underlying one-parameter beta distribution to model mergers, the authors show how the parameter of this beta distribution ( α ) can be estimated from tree topology alone. They then devise a technique to carry out a Bayesian posterior joint inference of both α and the effective population size (which can vary with time) from a multifurcating genealogy. The techniques are applied to simulated data to test the performance of the method, and then on three real datasets—two involving infectious viral diseases and the third with Japanese sardine populations.

A review by Teo *et al.* [23] studies probabilistic models that extend calculations from trees to networks. Their focus is not on the evolution of the network itself, but rather on the evolution of a trait along a network; the information influencing the trait passes through nodes of the network via multiple paths. Determining the probability of a trait pattern on a network extends classic probabilistic calculations of trait patterns on a tree; the key idea in performing the extension is to consider the evolution of a trait along a fixed network as a belief propagation problem using ideas from the study of graphical models. Teo *et al.* consider many scenarios involving discrete and continuous traits, devoting attention both to the logic of the computations and to their use in parameter inference.

### (d) Applications of phylogenetic models in specific domains

Finally, five papers highlight the impact of the Yule model on different areas of biological application. Across biological fields, Yule-like models are commonly assumed as generative models for phylogenetic trees, enabling ‘phylodynamic analysis’ [24] (i.e. the quantification of population dynamics—such as speciation rates or transmission rates—based on the trees). The widespread use of such approaches started with the extension of the Yule model to incorporate death and incomplete present-day sampling into a birth–death model for phylodynamic analysis [25]. As discussed by Pennell & MacPherson [12], this framework became influential in macroevolutionary studies of present-day species. More recently, through the availability of sequentially sampled sequences from pathogens during epidemics, the birth–death model has been extended to sequential sampling [26]—a modelling feature that has also been adopted for modelling sequential fossil sampling in macroevolution [27].

In the area of macroevolution, do Rosario Petrucci *et al.* [28] employ the sampling-through-time generalization of the birth–death model (‘fossilized birth–death process’, FBD) for fossil and extant species data. The authors combine the FBD with the so-called state-dependent speciation and extinction process [29], where birth or death of a species depends on its trait, with all possible trait values being a finite set (often of size two, corresponding to a binary trait). In this way, different traits and thus species may carry a different fitness. The authors demonstrate that by using fossils in addition to extant species data, the accuracy of trait-dependent extinction rate estimates increases considerably, though challenges remain regarding spurious correlations of neutral traits and birth–death rates.

Veron *et al.* [30] highlight that although classic macroevolutionary models assume that speciation occurs instantaneously, it is more plausible that there is an initiation of speciation, and after some time, a completion of speciation—so that microevolutionary processes within populations influence macroevolutionary between-species processes. Building upon a protracted speciation model by Etienne & Rosindell [31] and Etienne *et al.* [32], the paper explores the implications of protracted speciation for the interpretation of speciation rates estimated from phylogenetic trees. Based on mathematically derived properties, the authors approximate the protracted birth–death model by a common time-varying birth–death process and analyse the corresponding speciation and extinction rates. They highlight that care must be taken when interpreting rates towards the present, where the most recent speciation events have not yet had the time to reach completion, and show that rates in the past are not primarily influenced by speciation completion rates. These results provide a potential explanation for the apparent lack of association between the speed at which populations acquire reproductive isolation and phylogenetic estimates of speciation observed in empirical data.

Koelle & Rasmussen [33] explore fitness variation of pathogen strains during an epidemic, adopting a framework similar to that of the macroevolutionary study of do Rosario Petrucci *et al.* [28]. For pathogens, fitness differences—varying birth and death rates across individuals—might not be attributable only to a few different states. Instead, each single mutation might have deleterious effects (increased death or decreased birth rates). Thus far, only methods considering a small number of mutations are available [34]. Koelle & Rasmussen [33] explore the robustness of the available neutral phylodynamic inference framework in the presence of a large number of possibly deleterious mutations. They simulate trees with fitness effects and apply a neutral phylodynamic model to infer underlying parameters such as the viral growth rate and the time of the root. For the scenarios investigated, these general epidemiological parameters are estimated reliably despite ignoring the fitness effects of mutations.

Finally, two papers employ the phylodynamic framework for novel biological applications. First, Zwaans *et al.* [35] use the birth–death framework to model single-cell division and death. In the cell biology field, the phylogenetic tree represents a tree of cell divisions and death rather than an evolutionary tree. Recent CRISPR-Cas9 technology introduces barcodes into a first cell, with the barcode accumulating random changes over time, enabling the reconstruction of these single-cell phylogenetic trees. Zwaans *et al.* [35] introduce a framework for the barcode evolution model, and they apply it, in combination with the classic birth–death model, to zebrafish data from early development.

In the second paper to employ the phylodynamic framework, Dumm *et al.* [36] improve phylogenetic inference of B cell trees in the setting of immunology. Again, birth corresponds to cell division and death to cell apoptosis. The authors introduce advances to GCtree, a phylogenetic inference tool that relies on abundances of sampled sequences. They focus on novel data structures, highlighting in a benchmarking study that computational runtime remains feasible.

## 3. Prospects

It is a curious feature of Yule’s ‘loafer of the world’ academic style in the early years of the field of statistics that several of his efforts, including early use of regression in social sciences [37], early use of statistical analysis to perform authorship attribution on texts [38] and of course, the birth process for evolutionary descent as celebrated in this volume [5], have eventually come to be recognized for anticipating large bodies of research—long after having been somewhat hidden in the relevant domains of application. Looking back to the fact that in the late 1800s and early 1900s, the early mathematics of trees developed separately from the early evolutionary biology, perhaps only a mathematician or statistician with a wide-ranging taste for scientific problems and a knack for applied intuition could have made the first indirect link between the two, even if the application area of evolutionary biology was not prepared to make use of the insight.

Since its development as a distinctive area in the late twentieth century, the mathematical modelling tradition in phylogenetics has produced rich bodies of work on mathematical theory, statistical inference methods and application in diverse biological settings. The themed collection finds that many of the issues whose rudiments can be seen in Yule’s early paper [12]—including the challenges of macroevolutionary parameter estimation and identifiability [20,28,30], the use of phylogenetic tree properties to make inferences about modes of diversification [15,21,33] and the interface of population processes with phylogenetics [22,23,30]—have finally risen to prominence in the field. The papers illustrate the impact of the birth process first used for macroevolution across many areas, including computer science and population genetics, among others [13,16], and its contributions to the fields of probability and statistics more generally [13,14]. The volume finds exciting trends in phenomena that extend well beyond the topics of the first several decades of phylogenetic modelling—including multifurcating trees [17,22], lineage-dependent diversification processes [28,33], networks [18,19,23] and the new data types of modern biology [13,33,35,36]. We hope that this themed collection will provide many nodes through which the information guiding the next generation of phylogenetic modelling studies will pass as the network of modelling efforts continues into its second century.

## Data Availability

This article has no additional data.
